# Professor Gunning S. Bedford

**Published:** 1861-05

**Authors:** 


PROFESSOR GUNNING S. BEDFORD.—We are happy to see that the honor of a French translation has been extended to Professor Bedford's "Clinical Lectures on the Diseases of Females." Such a compliment must be highly gratifying to the distinguished author.

